# Can we abandon foregut exclusion for an ideal and safe metabolic surgery?

**DOI:** 10.3389/fendo.2022.1014901

**Published:** 2022-11-10

**Authors:** Jason Widjaja, Yuxiao Chu, Jianjun Yang, Jian Wang, Yan Gu

**Affiliations:** ^1^ Department of General Surgery, Fudan University Affiliated Huadong Hospital, Shanghai, China; ^2^ Department of Gastrointestinal Surgery, Affiliated Hospital of Xuzhou Medical University, Xuzhou, Jiangsu, China

**Keywords:** foregut hypothesis, micronutrient, type-2 diabetes, bariatric surgery, metabolic surgery

## Abstract

Foregut (foregut exclusions) and hindgut (rapid transit of nutrients to the distal intestine) theories are the most commonly used explanations for the metabolic improvements observed after metabolic surgeries. However, several procedures that do not comprise duodenal exclusions, such as sleeve with jejunojejunal bypass, ileal interposition, and transit bipartition and sleeve gastrectomy were found to have similar diabetes remission rates when compared with duodenal exclusion procedures, such as gastric bypass, biliopancreatic diversion with duodenal switch, and diverted sleeve with ileal interposition. Moreover, the complete exclusion of the proximal intestine could result in the malabsorption of several important micronutrients. This article reviews commonly performed procedures, with and without foregut exclusion, to better comprehend whether there is a critical need to include foregut exclusion in metabolic surgery.

## Introduction

**Figure 1:**
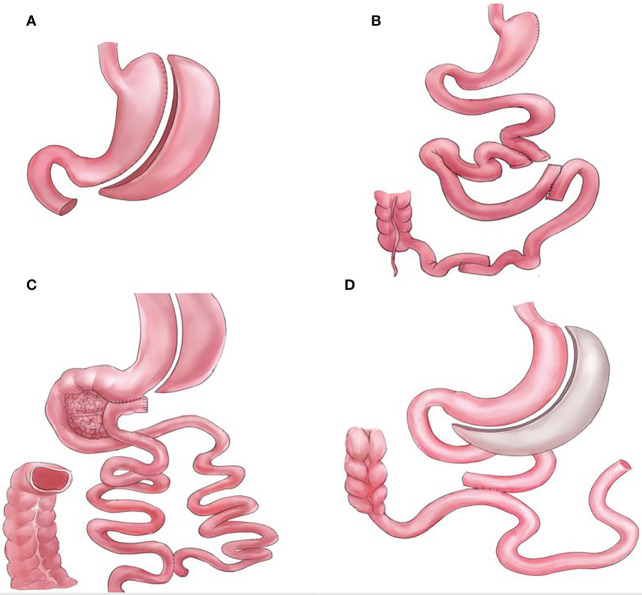
Graphical illustrations for procedures that do not bypass the foregut, **(A)** standalone sleeve gastrectomy (SG), **(B)** sleeve with ileal interposition (SG-II), **(C)** sleeve with transit bipartition (SG-TB), and **(D)** sleeve with jejunojejunal bypass (SG-JJB).

**Figure 2:**
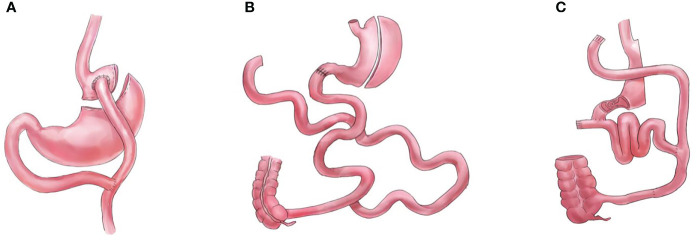
Graphical illustrations for procedures that completely bypass the foregut, **(A)** gastric bypass (GB), **(B)** biliopancreatic diversion with duodenal switch (DS), and **(C)** diverted sleeve with ileal interposition (DSG-II).

Bariatric and metabolic surgeries have resulted in significant improvements and remissions in type 2 diabetes mellitus and other metabolic comorbidities (1, 2). However, the mechanisms underlying these effects remain unclear. The foregut (proximal intestine exclusions) and hindgut theories are the classic and most commonly used explanations for the resolution of type 2 diabetes mellitus observed after metabolic surgeries (3). While the hindgut theory (rapid transit of nutrients to the distal intestine) has been widely accepted, the foregut theory is not (4, 5). Several procedures that do not comprise duodenal exclusions, such as sleeve with jejunojejunal bypass/SG-JJB, sleeve with ileal interposition/SG-II, sleeve with transit bipartition/SG-TB, and standalone sleeve gastrectomy/SG (**Figure 1**), have similar diabetes remission outcomes when compared with procedures comprising duodenal exclusions, such as gastric bypass/GB, biliopancreatic diversion with duodenal switch/DS, and diverted sleeve with ileal interposition/DSG-II (**Figure 2**) (6–13). Furthermore, the complete exclusion of the proximal intestine may result in significant micronutrient malabsorption. Thus, while being a safe metabolic procedure, the need for foregut exclusion to achieve ideal metabolic outcomes is questioned.

In this article, we briefly review the commonly performed metabolic procedures with and without foregut exclusion, with the hope to create a research direction to further comprehend the mechanisms of bariatric and metabolic surgeries.

## Foregut hypothesis

The foregut hypothesis is one of the classic explanations for the diabetes remission observed after bariatric surgery. This hypothesis proposes that the exclusion of the proximal small intestine (duodenum and proximal jejunum) from the transit of nutrients may prevent the secretion of a “factor” that promotes insulin resistance and type 2 diabetes mellitus (3, 14, 15). However, the foregut hypothesis fails to explain how several other bariatric procedures that did not comprise duodenal exclusion, such as SG-JJB, SG-II, SG-TB, and standalone SG, still achieved excellent diabetes remission results (6, 7, 10–12). Furthermore, we are yet to identify the foregut “factor” that affects the glucose homeostasis. If the exclusion of the foregut is not required to achieve metabolic improvements, then complete exclusion of the foregut may be abandoned to achieve the ideal metabolic procedure.

## Importance of foregut inclusion in bariatric and metabolic surgeries

There are significant drawbacks of excluding the foregut from nutrient transit. The proximal intestine is a major site of absorption of several important vitamins and micronutrients. For example, the risk of vitamin B12 deficiency is higher following GB than after SG and is attributable to the duodenal exclusion in gastric bypass (16). Procedures that exclude the proximal intestine, where most iron absorption occurs, are expected to increase the risk of iron deficiency (17). Reduced calcium absorption and vitamin D deficiency were also frequently observed in procedures that excluded the proximal intestine, which has the highest concentration of calcium transporters (18). Furthermore, the proximal intestine is also indirectly involved in the absorption of liposoluble vitamins and micronutrients. Procedures that bypass the proximal intestine result in the reduction of pancreatic enzyme secretion and alteration in bile salts, leading to alterations in fat assimilation (19–21).

## Procedures that completely bypass the foregut

GB, DS, and DSG-II are the procedures that are normally performed according to the foregut hypothesis (bypassing the foregut). These procedures can incorporate modifications of the “one-anastomosis reconstruction”; however, such modifications do not alter the foregut exclusion components.

Multiple randomized controlled trials (RCT) have compared the efficacy of GB with procedures that do not comprise foregut exclusion. A meta-analysis of RCTs comparing the outcomes of GB and SG found that GB resulted in a superior loss of body mass index (BMI), which persisted at 3 years postoperatively (22). Interestingly, the study did not find differences in diabetes remission, hemoglobin A1c (HbA1c) level, and homeostatic model assessment of insulin resistance levels. Similarly, three RCTs (Stampede, SM-BOSS, and SLEEVE-PASS trials) found no difference between GB and SG regarding HbA1c levels and rate of diabetes remission at 5 years postoperatively (12, 13, 23). However, GB is associated with a higher risk of nutritional deficiencies than SG (16, 24). Studies have reported that reconnecting the foregut back to the configuration can solve the malnutrition issue in GB without compromising the bariatric and metabolic outcomes (25, 26).

Another classic bariatric and metabolic procedure is DS, which is regarded as the most effective procedure (27). An RCT reported that at 5 years postoperatively, DS resulted in superior weight loss and glucose improvements compared to those of GB; however, DS is associated with more nutritional complications (28). Long-term studies (10 years) have also reported the nutritional issues associated with DS, particularly with fat-soluble vitamins (29, 30). Thus, although the effectiveness of DS is undisputed, it is not commonly performed owing to the high prevalence of complications (2, 31).

DSG-II and SG-II are among the most complex procedures, as they require more anastomosis than most metabolic surgeries (32). Unlike DSG-II, which bypasses the foregut and creates malabsorption, the SSG-II procedure ensures that there is no malabsorption. However, limited data are available regarding the prevalence of nutritional deficiency between the DSG-II and SG-II procedures. However, DSG-II and SG-II resulted in similar glucose control improvements, which further questions the need for foregut exclusion (7, 33, 34). Ileal interposition procedures are not commonly performed because of their complexity and the need for a high number of anastomoses, and because a higher number of mesenteric defects are created in these procedures (32).

## Procedures that do not bypass the foregut

SG, SG-TB, SG-II, and SG-JJB are the procedures that are not normally performed according to the foregut hypothesis. Similar to the GB and DS, some of these procedures can incorporate modifications of the “one-anastomosis reconstruction”; however, such modifications do not alter the foregut inclusion components.

SG can be considered the foundation of most bariatric and metabolic surgeries, as many of the procedures consist of SG. Standalone SG is currently the most commonly performed procedure worldwide, with excellent results (2, 31). As mentioned previously, when compared with GB, SG was found to be comparable regarding metabolic improvements as well as having a lower risk of nutritional deficiency (12, 13, 16, 23, 24). However, in the longer term, SG is complicated by several issues, such as weight regain, diabetes relapse, and reflux (35). Therefore, revisional surgery after SG is becoming a common practice.

SG-TB is a procedure that has been gaining significant attention in recent years (particularly its one-anastomosis form, the single-anastomosis sleeve ileal bypass/SASI). Proposed as a modification of the DS procedure, SG-TB eliminates the need to completely bypass the duodenum. The 5 year result of SG-TB was reported to be 74% excess BMI loss and 86% diabetes remission (5). SG-TB has been shown to have comparable weight loss and diabetes remission results, while having lesser risks for nutritional deficiencies when compared to GB (11, 36). When compared with DS, SG-TB was reported to have lesser weight loss results; however, there was no difference in the rate of diabetes remission (10). The authors further noted that SG-TB showed real benefits in reducing the side effects and malnutrition risks compared with DS (10). When compared with DSG-II, SG-TB showed similar weight loss and diabetes remission results; however, the differences in nutritional deficiencies between the two procedures has not been reported (9).

The SG-II procedure has been described and discussed in the previous section. Moreover, in the case of severe malnutrition after the DS procedure, conversion to SG-II (without bypassing the foregut) solved the malnutrition issues without compromising the bariatric and metabolic results (37). In conclusion, although more studies are needed, the SG-II and DSG-II demonstrated comparable weight loss and diabetes remission results; thus, questioning the need for foregut exclusion.

Other procedures that do not bypass the foregut have been reported. SG-JJB was reported to have comparable weight loss and diabetes remission rates to GB (6). A rodent model of jejunal-ileal loop bipartition has also been described, showing its effectiveness in improving glucose control (38).

SG-TB, SG-JJB, and SG-II procedures are still lacking comparative studies as well as RCTs; thus, further studies are warranted in this regard in the future. However, the results to date have been promising with regard to the notion of abandoning foregut exclusion.

## Mechanisms related to the effect of bariatric surgery

Glucagon-like peptide-1 (GLP-1) is secreted by intestinal enteroendocrine L-cells and several brain cells in the brainstem following food consumption (39). GLP-1 is an incretin having the ability to enhance insulin secretion. In the brain and the stomach, GLP-1 also can promote satiety, reducing food intake. Per the hindgut theory (rapid transit of nutrients to the distal intestine), bariatric procedures that resulted in the rapid transient of nutrients showed significantly elevated GLP-1 levels following food consumption (39). A recent meta-analysis reported that postprandial GLP-1 levels were also increased following SG, possibly due to increased gastric emptying (40).

Similarly, peptide YY (PYY) was also reported to be elevated in bariatric procedures with or without duodenal exclusion (40). PYY is also secreted by the L-cells and has the ability to reduce appetite and promote satiety.

After bariatric surgery, several studies have shown that the increased level of bile acids can promote insulin secretion, increase energy expenditure, and alter the gut microbiota (41). Bile acids play a role in metabolic regulation mediated through several receptors, such as the farnesoid X receptor (FXR) and G protein-coupled bile acid receptor 1 (also known as TGR5) (41). Stimulation of FXR in insulin-resistant obese mice was shown to reduce hyperinsulinemia and improved glucose control (42). In response to bile acids, TGR5 activation promotes GLP-1 secretion in animal and human studies (43, 44). Increased bile acids can increase energy expenditure through TGR5 in the skeletal muscle and brown adipose tissue (45, 46). Bile acids and gut microbiota are affected and altered by bariatric surgery. Increased bile acid concentrations can kill and promote certain gut bacteria strains (41).

Individuals with obesity exhibit an altered gut microbiota as compared to lean controls, comprising of a decline in Bacteroidetes and an increase in Firmicutes in obese individuals (47). On the other hand, bariatric surgery resulted in the alteration of gut microbiota composition (decrease of Firmicutes/Bacteroidetes ratio), which contributes to fat mass regulation and reduced utilization of carbohydrates as energy fuel (48).

Several studies have shown that bariatric surgery induces changes in adipose tissue and improves systemic inflammation (49). Bariatric surgery induces changes in the levels of several microRNAs from the adipocyte-derived exosomes, which are correlated to the improved insulin signaling following the surgery. Several inflammatory factors, such as C-reactive protein, tumor necrosis factor-α, and interleukin-6, the hallmark for the initiation of insulin resistance, were also reduced following bariatric surgery.

Several other hypotheses explaining the mechanisms of bariatric surgery exist (41). However, all of these hypotheses can be justified through the anatomical changes leading to the distal intestine or as changes in general after bariatric surgery, further questioning the foregut hypothesis.

## Discussion

The era of bariatric and metabolic surgeries has been evolving continuously. In the past, malabsorption and restriction were the primary targets of bariatric surgery for achieving an ideal healthy weight (50). However, in recent years, the era of pure metabolic surgery has been initiated, focusing on improving the metabolic potency of bariatric surgery, hence the name “metabolic surgery” (51–53). To improve the metabolic potency of a procedure, we must comprehend the mechanism of the metabolic improvements observed following metabolic procedures. However, metabolic surgery appears to have a highly complex mechanism, and more time may be needed to better understand it. Moreover, recent surgical innovations have provided us with the knowledge that could be used to improve the safety of metabolic surgery.

Classic and significant metabolic procedures, such as GB and DS, resulted in excellent metabolic outcomes (54–57). However, they also resulted in unwanted effects, such as excessive nutrient malabsorption (58). In contrast, several metabolic procedures (such as SG-JJB, SG-II, SG-TB, and SG) that maintain the foregut (either completely or partially) have been demonstrated to have efficacy that is not inferior to foregut exclusion procedures (6–13). It is imperative to acknowledge that the SG procedure has been the most performed bariatric procedure worldwide in recent years, surpassing RYGB (31). Furthermore, several RCTs have shown that SG (without duodenal exclusion) could result in comparable bariatric and metabolic outcomes compared to RYGB (with duodenal exclusion) (12, 13, 23).

Although procedures such as SG-JJB, SG-II, and SG-TB, differ in intestinal reconfiguration, they have common consequences: 1) foregut inclusion and 2) expediting nutrient flow to the distal intestine (hindgut theory). It has been recently proposed that foregut exclusions may not be necessary as long as strong stimulus to the ileum is provided (59). However, we need better comparative studies to understand not only the metabolic efficacy but also the safety of these foregut inclusion procedures.

With the foregut hypothesis being the focus of this article, it is imperative to discuss the use of duodenal-jejunal bypass liner as a treatment alternative for metabolic diseases. While it has been reported that the duodenal-jejunal bypass liner resulted in significant improvements in type 2 diabetes, the underlying mechanisms remain elusive (60). In contrast to foregut exclusion, a previous study showed that preserving foregut transit in GB and DS models still resulted in significant weight loss and glucose control improvements (25, 26, 37). Therefore, these findings created another notion, “Is bypassing the foregut necessary? Or as long as there is enough exclusion, regardless of the site of exclusion, would we still observe excellent metabolic improvements?” The goal of bariatric and metabolic surgery should be to improve the patients’ quality of life as well as improving their weight status and comorbidities, i.e. not to focus solely on the weight loss outcomes.

In conclusion, with the available studies, we cannot deny the credibility of foregut exclusion for excellent metabolic outcomes. However, the idea of abandoning complete foregut exclusion has some credibility, and more comparative studies are needed to prove this idea. Such studies should focus mainly on whether: 1) foregut inclusion resulted in non-inferior metabolic outcomes than after foregut exclusion and 2) foregut inclusion delivers better safety regarding micronutrient malabsorption than that following foregut exclusion.

## Author contributions

JaW and YG propose the topic and design the manuscript flows. JaW and JY draft the manuscript. YC and JiW collect and analyse the materials for the manuscript. JW, YC, JY, JiW, and YG reviewed the manuscript. All authors contributed to the article and approved the submitted version.

## Conflict of interest

The authors declare that the research was conducted in the absence of any commercial or financial relationships that could be construed as a potential conflict of interest.

## Publisher’s note

All claims expressed in this article are solely those of the authors and do not necessarily represent those of their affiliated organizations, or those of the publisher, the editors and the reviewers. Any product that may be evaluated in this article, or claim that may be made by its manufacturer, is not guaranteed or endorsed by the publisher.
